# IgG4-Related Membranous Nephropathy After COVID-19 Vaccination: A Case Report

**DOI:** 10.7759/cureus.56028

**Published:** 2024-03-12

**Authors:** Tomohito Mizuno, Yoko Endo, Atsushi Suzuki, Masashi Suzuki

**Affiliations:** 1 Department of Internal Medicine, Division of Nephrology, Tokyo Yamate Medical Center, Tokyo, JPN; 2 Department of Pathology, Tokyo Yamate Medical Center, Tokyo, JPN

**Keywords:** covid-19 vaccine, severe acute respiratory syndrome coronavirus 2, membranous glomerulopathy, igg4-related kidney disease, igg4-related disease

## Abstract

Although immunoglobulin G4 (IgG4)-related kidney diseases are typically characterized by tubulointerstitial nephritis with abundant infiltration of IgG4-positive plasma cells and fibrosis, there have been relatively rare cases of IgG4-related glomerulonephritis. Several cases of IgG4-related disease (IgG4-RD) following coronavirus disease 2019 (COVID-19) mRNA vaccination have been reported. However, there are no reports of IgG4-related glomerulonephritis following COVID-19 vaccination. Herein, we present a case of IgG4-related membranous nephropathy (MN) occurring after COVID-19 vaccination.

A 69-year-old Japanese male presented to our hospital with edema that started the day after his second COVID-19 vaccination. The patient exhibited nephrotic syndrome and was diagnosed with MN based on the results of a kidney biopsy. Although serum IgG4 levels were elevated to 946 mg/dL, no evidence of organ involvement suggestive of IgG4-RD was observed. Treatment with prednisolone and cyclosporine resulted in complete remission, and immunosuppressive agents were tapered. However, one month after discontinuing the immunosuppressive agents, the patient was readmitted with swelling around the submandibular glands and exertional dyspnea. Serum IgG4 level was markedly elevated at 2,320 mg/dL, and computed tomography revealed submandibular gland swelling and thickening of the interlobular septum and bronchovascular bundles in both lungs. The patient was diagnosed with IgG4-RD based on elevated serum IgG4 levels and infiltration of IgG4-positive plasma cells in the submandibular gland biopsy. Upon resuming treatment with prednisolone, the symptoms attributed to IgG4-RD improved within a few days. In cases of nephrotic syndrome following COVID-19 vaccination, it may be advisable to conduct detailed examinations to assess the possibility of the development of IgG4-RDs.

## Introduction

Immunoglobulin G4-related disease (IgG4-RD) is characterized by elevated serum IgG4 levels, multiple organ enlargement, and abundant infiltration of IgG4-positive plasma cells with fibrosis [1]. IgG4-RD first recognized as autoimmune pancreatitis, is a disease entity of systemic fibroinflammatory disorder that manifests asynchronously in multiple organs, including the salivary glands, lacrimal glands, kidneys, liver, and lungs [2]. In IgG4-related kidney disease (IgG4-RKD), the typical histopathological finding is plasma cell-rich tubulointerstitial nephritis (TIN), with relatively few cases showing glomerulonephritis [3].

Efficient vaccines against the severe acute respiratory syndrome coronavirus type 2 (SARS-CoV-2) are widely distributed worldwide and contribute to a reduction in the severity and mortality of coronavirus disease 2019 (COVID-19) [4]. However, reports of various vaccine-related adverse reactions are increasing. While many adverse reactions to COVID-19 vaccines are mild to moderate and naturally resolve, several cases require intensive care or result in fatalities [4]. The development or exacerbation of autoimmune diseases caused by COVID-19 vaccines is an important adverse reaction requiring specialized treatment [5]. While the mechanism remains incompletely understood, it underscores the complexity of the disease-vaccine interactions. Although several cases of IgG4-RD following COVID-19 vaccination have been reported, to the best of our knowledge, there are no reports of new-onset glomerulonephritis associated with IgG4-RD. Herein, we present a case of IgG4-related membranous nephropathy (MN) after vaccination with the Pfizer/BioNTech BNT162b2 COVID-19 vaccine.

## Case presentation

A 69-year-old Japanese male presented to his primary care physician with complaints of bilateral lower leg swelling and foamy urine the day after receiving a second dose of the BNT162b2 COVID-19 vaccine, specifically on the 22nd day after the first vaccine dose. Urine analysis detected proteinuria for the first time. The patient did not show any albuminuria during his regular check-up three months prior to presentation. The patient had a 10-year history of diabetes mellitus during which there was no evidence of diabetic nephropathy or retinopathy. His diabetes was generally well managed with dipeptidyl peptidase-4 inhibitors and biguanides, maintaining a hemoglobin A1c level < 7%.

Eight days later, he was referred to our hospital and was admitted with a diagnosis of nephrotic syndrome. Upon admission, his height was 167 cm and weight was 58.4 kg, showing a 5 kg weight gain compared with the previous month. His blood pressure was 127/80 mmHg, pulse rate was 85 beats per minute, and body temperature was 36.9℃. Physical examination revealed bilateral lower leg edema but no other abnormalities.

The laboratory data at first admission are shown in Table 1. Laboratory investigations revealed that the serum albumin decreased to 1.3 mg/dL and the urine protein increased to 5.8 g/gCr, indicative of nephrotic syndrome. The serum creatinine level was mildly elevated (1.3 mg/dL). Serum transaminase and amylase levels were normal. Serum IgG was 1,754 mg/dL, with serum IgG4 elevated to 946 mg/dL. Serum complement levels were normal, and hepatitis B surface antigen and hepatitis C virus antibody tests were negative. Tests for autoimmune antibodies, including MPO-ANCA, PR3-ANCA, and antinuclear antibodies, were negative.

**Table 1:** Laboratory data of the patient. Abbreviations: GFR, glomerular filtration rate; MPO-ANCA, myeloperoxidase-anti-neutrophil cytoplasmic antibodies; PR3-ANCA, proteinase-3-anti-neutrophil cytoplasmic antibodies; SS-A, Sjӧgren’s related-antigen A; SS-B, Sjӧgren’s related-antigen B; ACE, angiotensin-converting enzyme; PLA2R, phospholipase A2 receptor; NAG, N-acetyl-β-D glucosaminidase

	On 1^st^ admission	On 2^nd^ admission	Reference range
Biochemistry/immunology			
Total protein (g/dL)	5.2	9.5	6.5–8.0
Serum albumin (g/dL)	1.3	3.4	3.9–4.9
Aspartate aminotransferase (U/L)	21	18	10–33
Alanine aminotransferase (U/L)	13	12	4–30
Alkaline phosphatase (U/L)	59	47	38–113
Total bilirubin (mg/dL)	0.5	0.4	0.2–1.2
Amylase (U/L)		59	30–120
Blood urea nitrogen (mg/dL)	22	33	8–20
Serum creatinine (mg/dL)	1.30	1.37	0.65–1.07
Estimated GFR (ml/min/1.73m^2^)	43.2	40.4	
Serum sodium (mEq/L)	139	134	135–145
Serum potassium (mEq/L)	4.5	5.4	3.4–5.0
Serum chloride (mEq/L)	104	102	98–108
Serum calcium (mg/dL)	8.5	9.2	8.5–10.3
C-reactive protein (mg/dL)	0.1	3.7	0–0.4
Hemoglobin A1c (%)	6.6	6.3	4.6–6.2
Immunoglobulin G (mg/dL)	1754	3972	870–1,700
Immunoglobulin A (mg/dL)	290	2320	110–410
Immunoglobulin M (mg/dL)	55	73	33–190
Immunoglobulin G4 (mg/dL)	946	2320	11–121
C3 (mg/dL)	109	62	86–160
C4 (mg/dL)	26	5	17–45
CH50 (U/mL)	45.4	26.0	25.0–48.0
Antinuclear antibody (titer)	1:40	1:40	< 40
MPO-ANCA (U/mL)	< 1.0	< 1.0	< 3.5
PR3-ANCA (U/mL)	< 1.0	< 1.0	< 3.5
Anti-SS-A antibody		Negative	
Anti-SS-B antibody		Negative	
ACE (U/mL)		7.9	7.7–29.4
Hepatitis B surface antigen	Negative		
Hepatitis C virus antibody	Negative		
Anti-PLA2R antibody	Negative		
Complete blood cell count			
White blood cell (/µL)	4280	3820	3,500–9,000
Neutrophil (%)	36.5	82.0	37.0–72.0
Lymphocyte (%)	28.0	13.5	19.0–49.0
Monocyte (%)	12.9	3.5	2.0–11.0
Eosinophil (%)	18.9	1.0	0–5.0
Red blood cell (× 10^4^/µL)	475	405	410–530
Hemoglobin (g/dL)	15.5	12.3	14.0–18.0
Hematocrit (%)	45.3	37.2	40.0–55.0
Platelet (× 10^4^/µL)	21.2	26.0	12.0–36.0
Urinalysis			
Protein	4+	-	
Occult blood	±	-	
Red blood cell count (/HPF)	1–4	1–4	
Protein content (g/gCr)	5.8	0.2	
NAG (U/L)	58.2	20.9	< 11.2
β2 microglobulin (μg/L)	< 100	< 100	< 200

**Figure 1:**
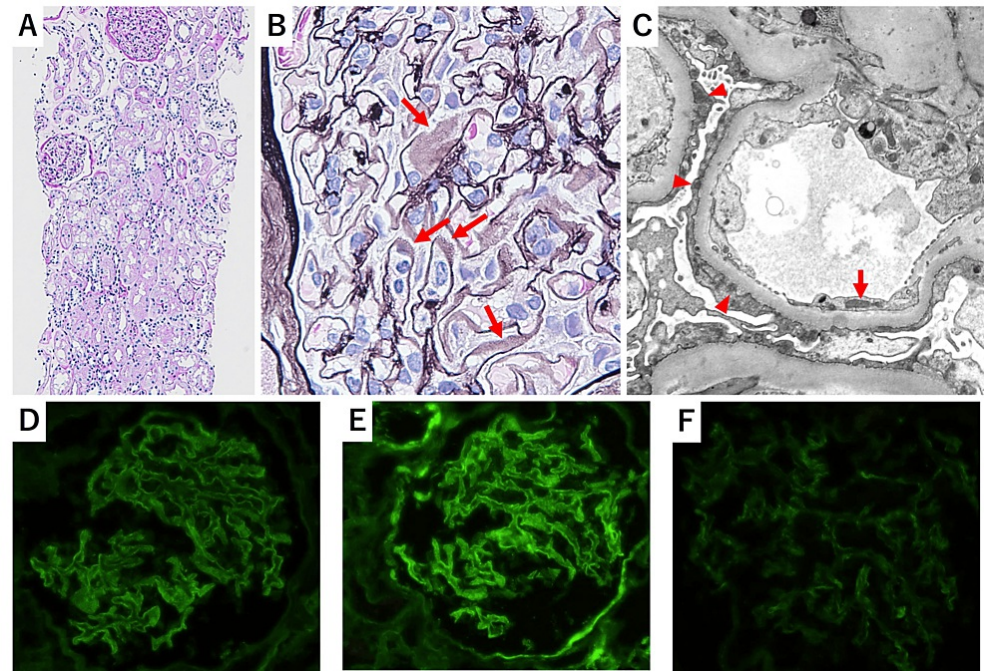
Histological examination of the kidney biopsy. (A, B) Light microscopy analysis of the kidney biopsy. (A) Periodic acid-Schiff stain: Inflammatory cell infiltration including plasmacyte or interstitial fibrotic change was not observed (400×). (B) Periodic acid-methenamine-silver stain: Pinholes of GBM were observed (arrow) (400×). (C) Electron microscopy revealed finely granular dense deposits in the subepithelial space (arrowhead) and in the subendothelial space (arrow). GBM was diffusely thickened. Immunofluorescence analysis showed granular IgG and C3 deposition along the GBM and weak C1q deposition (200×). (D) IgG, (E) C3, (F) C1q.

A kidney biopsy was performed to determine the cause of renal dysfunction (Figures 1A-1F). Pinholes of the glomerular basement membrane (GBM) were observed in periodic acid-methenamine-silver staining and subepithelial deposition in Masson staining. No inflammatory cell infiltration or interstitial fibrotic changes were observed. The renal tubular epithelium showed foamy degeneration reflecting proteinuria. Immunofluorescence microscopy revealed granular deposition of IgG and C3 along the glomerular capillary walls and weak C1q deposition. Electron microscopy revealed dense deposits in subepithelial and subendothelial spaces. The GBM was diffusely thickened, and the mesangial matrix increased. The microvillous transformation of podocytes was also observed. Based on these findings, the patient was diagnosed with stage I MN and early-stage diabetic nephritis. Although serum IgG4 levels were elevated, lymphoplasmacytic infiltrates rich in IgG4-positive plasma cells, which are characteristic of IgG4-RKD, were not observed. Serum phospholipase A2 receptor (PLA2R) antibodies tested negative. We considered secondary MN based on renal histological findings and the negative result for PLA2R antibodies; however, there was no evidence of secondary MN causes such as infections or malignancy.

**Figure 2:**
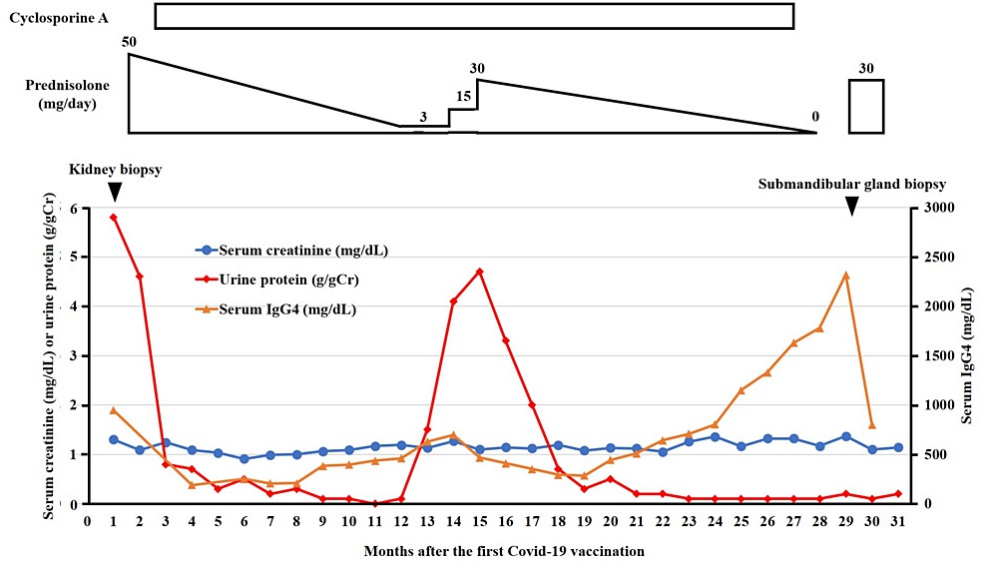
Clinical course after the coronavirus disease 2019 (COVID-19) mRNA vaccination. IgG4, immunoglobulin G4.

The patient was initiated on treatment with an initial dose of 0.8 mg/kg/day (50 mg/day) of prednisolone. Cyclosporine was added to the prednisolone regimen after four weeks because proteinuria persisted despite prednisolone therapy. Subsequently, proteinuria gradually decreased, leading to complete remission (Figure 2).

Approximately one year after the initiation of treatment, the patient experienced a single relapse of nephrotic syndrome while tapering prednisolone. Prompt improvement was achieved by increasing the prednisolone dose to 30 mg/day. Subsequently, there was no further relapse and the patient maintained complete remission. Therefore, immunosuppressive therapy with prednisolone and cyclosporine was discontinued approximately two years after the start of treatment.

**Figure 3:**
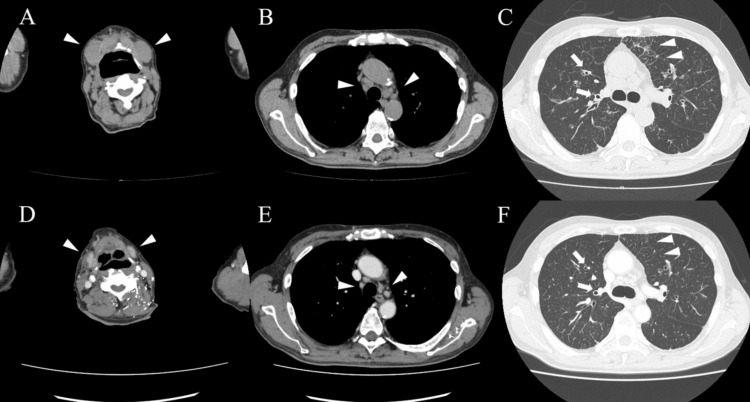
Computed tomography findings before and after resuming prednisolone. (A–C) Unenhanced computed tomography (CT) findings before resuming prednisolone. CT of the neck shows the swelling of bilateral submandibular glands (arrowhead in A). CT of the chest shows the enlarged mediastinal lymph nodes (arrowhead in B), the interlobular septum thickening (arrowhead in C), and the bronchovascular bundle thickening (arrow in C). (D–F) Contrast-enhanced CT findings 2 weeks after resuming prednisolone. CT of the neck shows improvement in both submandibular gland enlargement (arrowhead in D). CT of the chest shows improvement in the mediastinal lymph nodes enlargement (arrowhead in E), improvement in the interlobular septum thickening (arrowhead in F), and bronchovascular bundle thickening (arrow in F).

**Figure 4:**
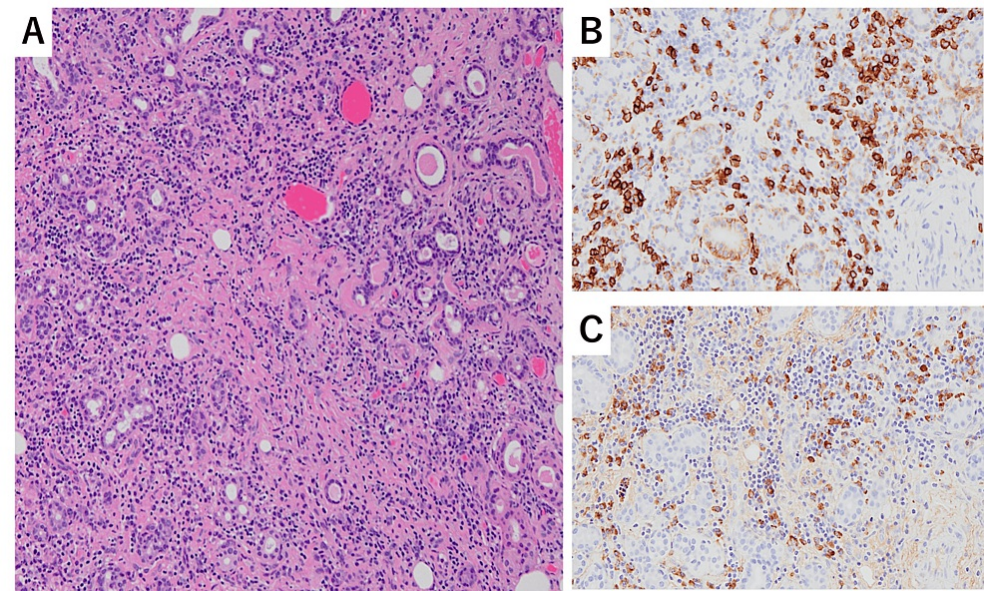
Light microscopy of the submandibular gland biopsy. (A) Hematoxylin-eosin stain: Infiltration of a large number of inflammatory cells, predominantly lymphocytes and plasma cells, accompanied by fibrous tissue proliferation. The acini and the ducts are destroyed (100×). (B, C) Immunohistochemistry showed IgG4-positive/CD138-positive cell ratio exceeding 40% (200×). (B) CD138, (C) IgG4

Approximately two weeks after discontinuing the immunosuppressive therapy, the patient experienced exertional dyspnea and swelling around the jaw, which gradually worsened and led to readmission to our hospital four weeks later. Physical examination upon readmission revealed swelling in the bilateral submandibular regions. Laboratory data on the second admission are shown in Table 1. Significant elevations in serum IgG and IgG4 levels along with hypocomplementemia were observed. Although the serum creatinine level was mildly elevated to 1.37 mg/dL, there was no evidence of urinary protein or hematuria, and urine sediment examination revealed no abnormalities. Tests for autoimmune antibodies, such as MPO-ANCA, PR3-ANCA, antinuclear antibodies, anti-SS-A antibodies, and anti-SS-B antibodies, were negative. Whole-body computed tomography (CT) revealed bilateral swelling of the submandibular glands and thickening of the interlobular septum and bronchovascular bundles in both lungs (Figures 3A-3F). A submandibular gland biopsy revealed significant plasma cell infiltration, interstitial fibrosis, and acinar and ductal destruction. Immunohistochemical staining revealed IgG4-positive/CD138-positive cell ratios exceeding 40% (Figures 4A-4C), leading to the diagnosis of IgG4-RD.

Prednisolone was resumed at a dose of 0.6 mg/kg/day (30 mg/day), and symptoms such as exertional dyspnea improved within a few days. Furthermore, within two weeks, the swelling of both submandibular glands decreased, and the chest abnormalities observed on CT also improved. Without the emergence of new symptoms, the patient was discharged while taking 30 mg/day of prednisolone. One month after resuming prednisolone treatment, the serum IgG4 level decreased to 802 mg/dL, and the serum complement levels almost normalized. Subsequently, prednisolone was tapered by 5 mg every two weeks until reaching 10 mg/day. At the final visit to our hospital two months after discharge, the serum IgG4 level decreased to 614 mg/dL, the serum complement levels were normal, and proteinuria was not observed. Additionally, there were no other findings suggestive of recurrence of IgG4-RD. The plan was to taper the prednisolone dose in accordance with the treatment protocol for IgG4-RD and continue maintenance therapy with low-dose prednisolone.

## Discussion

IgG4-RKD is observed in approximately 15%-30% of IgG4-RDs [6]. Histologically, it typically presents as TIN with abundant plasma cell infiltration and fibrosis, also known as IgG4-TIN; however, cases of glomerulonephritis have also been reported [3]. Among the glomerulonephritis observed in IgG4-RDs, MN is the most common, accounting for approximately 7% of IgG4-TIN cases in a previous report [7]. However, the precise frequency of IgG4-related MN remains unclear because it has been also reported that 38% of IgG4-related MN cases do not involve TIN [8]. The onset timing of IgG4-related MN varies; while most IgG4-related MN cases develop simultaneously with or several years after the involvement of other organs, rare cases precede manifestations in other organs [8]. In cases of IgG4-related MN without the involvement of other organs, it is difficult to distinguish between primary MN and IgG4-related MN at the onset. As a result, as observed in the present case, a diagnosis of IgG4-related MN may be diagnosed during the treatment course of the primary MN. Our patient did not fulfill the diagnostic criteria for IgG4-RD at the time of MN diagnosis. However, considering that the serum IgG4 level was already elevated at the onset of MN and that symptoms of IgG4-RD are generally characterized by asynchronous manifestations in multiple organs, it was concluded that the MN in our case was a manifestation of IgG4-RD [7]. Considering the incidence of MN and IgG4-RDs, it is extremely rare for both to occur simultaneously.

IgG4-related MN, in which autoantigens associated with MN, such as PLA2R and thrombospondin type-1 domain-containing 7A (THSD7A), have been reported [9,10]. While these autoantigens typically test positive in primary MN, some cases of apparent secondary MN, such as those with hepatitis B or hepatitis C virus infection, are also positive for these autoantigens [11]. There is an ongoing debate regarding whether these cases represent true secondary MN or merely the coexistence of primary MN and the causes of secondary MN [11].

The standard treatment for IgG4-related MN remains to be established, but it often requires immunosuppressants in addition to glucocorticoids because of its poor steroid responsiveness compared to IgG4-TIN [8]. Similar to our case, a previous study reported that a combination of glucocorticoids and cyclosporine was effective for IgG4-related MN. In contrast, another study reported that multitarget therapy with glucocorticoids, mycophenolate mofetil, and tacrolimus was more effective than combination therapy with glucocorticoids and cyclosporine in treating refractory IgG4-related MN [12,13].

Various autoimmune diseases have been reported following COVID-19 vaccination, including IgG4-RDs, although direct causality remains unproven [5]. Our case, which presented the development of IgG4-related MN shortly after COVID-19 vaccination, suggests a potential relationship between COVID-19 vaccination and the onset of IgG4-related MN. Masset et al. first reported the relapse of IgG4-TIN following COVID-19 vaccination, followed by reports of five cases of IgG4-RDs after COVID-19 vaccination [14-19]; four of the five cases were new-onset IgG4-RDs involving the pancreas, liver, pleura, or salivary glands, and one was a worsening case of IgG4-hepatopathy. To the best of our knowledge, this is the first report of IgG4-related MN following COVID-19 vaccination.

Additionally, new-onset and recurrent cases of MN following COVID-19 vaccination have been reported [20]. Distinguishing between IgG4-related MN without TIN from primary MN remains challenging. Furthermore, serum IgG4 levels are not usually measured at the time of diagnosing nephrotic syndrome. Therefore, among the reported cases of MN after the COVID-19 vaccination, there may have been undiagnosed cases of IgG4-related MN.

## Conclusions

Here, we report a case of IgG4-RD presenting with MN following COVID-19 vaccination, with subsequent relapse in the lungs and salivary glands after tapering prednisolone and cyclosporine. Kidney biopsy is a crucial diagnostic tool for kidney diseases; however, in IgG4-RKDs, there are rare cases where the diagnosis remains challenging even after the biopsy. Therefore, in cases of nephrotic syndrome following COVID-19 vaccination, particularly when accompanied by symptoms unrelated to renal involvement, considering the possibility of IgG4-RDs and pursuing further investigations, including serum IgG4 measurements and whole-body CT, may be warranted.
